# SCD leads to the development and progression of acute myocardial infarction through the AMPK signaling pathway

**DOI:** 10.1186/s12872-021-02011-8

**Published:** 2021-04-20

**Authors:** Lijie Wang, Fengxia Yu

**Affiliations:** 1 Department of Cardiology, The Fourth Affiliated Hospital of China Medical University, Shenyang, 110032 Liaoning China; 2 Department of General Practice, The Second Affiliated Hospital of Shenyang Medical College, Shenyang, China

**Keywords:** Gene expression, Acute myocardial infarction, Dysfunction module, Potential pathogenesis

## Abstract

**Background:**

Acute myocardial infarction (AMI) is myocardial necrosis caused by acute coronary ischemia and hypoxia. It can be complicated by arrhythmia, shock, heart failure and other symptoms that can be life-threatening. A multi-regulator driven dysfunction module for AMI was constructed. It is intended to explore the pathogenesis and functional pathways regulation of acute myocardial infarction.

**Methods:**

Combining differential expression analysis, co-expression analysis, and the functional enrichment analysis, a set of expression disorder modules related to AMI was obtained. Hypergeometric test was performed to calculate the potential regulatory effects of multiple factors on the module, identifying a range of non-coding RNA and transcription factors.

**Results:**

A total of 4551 differentially expressed genes for AMI and seven co-expression modules were obtained. These modules are primarily involved in the metabolic processes of prostaglandin transport processes, regulating DNA recombination and AMPK signal transduction. Based on this set of functional modules, 3 of 24 transcription factors (TFs) including NFKB1, MECP2 and SIRT1, and 3 of 782 non-coding RNA including miR-519D-3P, TUG1 and miR-93-5p were obtained. These core regulators are thought to be involved in the progression of AMI disease. Through the AMPK signal transduction, the critical gene stearoyl-CoA desaturase (SCD) can lead to the occurrence and development of AMI.

**Conclusions:**

In this study, a dysfunction module was used to explore the pathogenesis of multifactorial mediated AMI and provided new methods and ideas for subsequent research. It helps researchers to have a deeper understanding of its potential pathogenesis. The conclusion provides a theoretical basis for biologists to design further experiments related to AMI.

**Supplementary Information:**

The online version contains supplementary material available at 10.1186/s12872-021-02011-8.

## Background

Acute myocardial infarction (AMI) is the major cause of high mortality rates of cardiovascular disease in men and women. Chest compression is the common symptom in patients with acute myocardial infarction [1, 2]. Other symptoms include confusion, weakness, chest pain, difficulty breathing, and vomiting [3]. AMI is a subset of the acute coronary syndrome; it can be divided into ST-segment elevation myocardial infarction and non-ST-segment elevation myocardial infarction [4]. Myocardial ischemia–reperfusion injury in patients with ST-segment elevation myocardial infarction can induce no-reflow, leading to myocardial necrosis and apoptosis, and even poor prognosis [5]. Cardiovascular disease is a critical health problem in both developed and developing countries, especially in the United States. More than 6 million people were reported having AMI each year [6, 7]. Plaque rupture is the guiding cause of AMI. However, there are 6% to 12% of myocardial infarction patients with normal angiographic coronary artery [8]. Microvascular obstruction after AMI is closely related to adverse ventricular remodeling, arrhythmia and adverse clinical outcomes [9]. Influenza vaccination in patients with coronary artery disease has been shown to cause an increase in the morbidity and mortality of AMI [10]. Increasing research data suggested that myocardial infarction may be closely related to a variety of other diseases. On the one hand, according to Alpert et al. [11], myocardial infarction is usually the result of thrombotic coronary arterial obstruction caused by plaque rupture, ulceration, fissuring, or dissection. On the other hand, factors unique to diabetes contribute to myocardial infarction through increasing atherosclerotic plaque formation and thrombosis. AMI is also a critical cause of mortality in diabetic patients [12].

AMI can develop into cardiogenic shock and mechanical complications, and cardiogenic shock is the leading cause of in-hospital death in patients with AMI [13, 14]. Cardiogenic shock is a rapid progression of AMI and usually is a fatal complication [15]. Myocardial infarction is a crucial health problem and its mortality rate is more than double that of cancer. Some studies [16, 17] found that more than half of cardiovascular deaths are due to AMI. Although progress has been made in the early and long-term treatment of AMI, it is still the guiding cause of high morbidity and mortality in Western countries [18]. AMI may be a severe complication of many diseases including hypertrophic cardiomyopathy [19] and atrial fibrillation [20]. In the past 20 years, the short-term prognosis of patients with myocardial infarction has been steadily improved after the introduction of beta blockers, thrombolysis, and aspirin [21]. The common treatments including systemic thrombolysis and direct percutaneous coronary intervention are used for patients with AMI [22, 23]. A critical reduction in mortality of AMI has been achieved through aggressive strategies in early identification and intervention [24]. Therefore, it is imperative for medical scientists and biologists in various countries to explore the pathogenesis and treatment mechanism of AMI. In this study, based on a multifactorial mediated dysfunction module for AMI, a series of comprehensive analyses was performed to explore the relevant pathogenic genes of AMI, in order to identify the core signal transduction that triggered AMI. In general, the comprehensive strategy of this study provided not only new insights to the pathogenesis of AMI, but also abundant resources and guidance for biologists to design further experiments.

## Methods

### Differentially expressed genes (DEGs) analysis

An expression microarray dataset for the AMI disease sample of numbered GSE48060 was collected from the NCBI Gene Expression Omnibus (GEO) database [25]. DEGs analysis of two sets (normal-recurrent acute myocardial infarction, normal-no recurrent acute myocardial infarction) on the collected disease samples was performed and calculated using the limma package [26] in R. Finally, based on the combination of the two sets of genes, 4551 DEGs was obtained to construct a related expression matrix of AMI.

### Co-expression analysis identifies relevant functional modules

The weighted gene co-expression network analysis (WGCNA) [27] was used to analyze the gene expression profile matrices of the 4551 DEGs, and a gene module for synergistic expression was found. The correlation coefficient weighting value was used, which is the gene correlation coefficient to the power of N. Then, the correlation coefficient between any two genes was calculated. The connections between genes in the network are subject to scale-free network distribution, making the algorithm more biologically meaningful. A hierarchical clustering tree was constructed by correlation coefficients between genes. The different branches of the clustering tree indicated different gene modules and different colors indicated different modules. Seven co-expression modules were extracted, which were identified as essential dysfunctional modules of AMI.

### Functional enrichment analysis to identify dysfunction modules

Exploring the function of genes and their involvement in signal transductions is an effective means of studying the molecular mechanisms of disease. The functions and pathways involved in the module gene can characterize the dysfunction mechanism of the module during the AMI process. For the genes of the dysfunction module, functional enrichment analysis was performed using clusterProfiler package [28] in R language to the Go functions (*p*-value cutoff = 0.01, q-value cutoff = 0.01) and the Kyoto Encyclopedia of Genes and Genomes (KEGG) pathways (*p*-value cutoff = 0.05, q-value cutoff = 0.2). According to the functions and pathways which were involved in the module gene, it was identified as a related dysfunction module of AMI. GlueGO in Cytoscape [29] software was performed to functions and pathways for each module, to build corresponding function and access networks. Next, it was identified that the proportion of modules participating in the corresponding functions and pathways.

### Identification of transcription factors and ncRNA regulation of modules

All human transcription factors (TFs) target data were downloaded from the TRRUST v2 database [30], 26 interaction pairs of 24 TFs were obtained. Human ncRNA-mRNA data (score > 0.5) were downloaded in a RAID 2.0 database [31], and 1239 interaction pairs involving 782 ncRNA was obtained. To identify the regulatory effects of these TFs and ncRNA on the module, a pivot analysis based on these interaction data was performed. Pivot analysis refers to the search for a driver pair with at least two pairs of modules in a target pair. According to the hypergeometric test, it was calculated that the significance of the interaction between the drivers and the modules. It was screened TFs with a *p*-value < 0.01 and ncRNA as the pivot of the essential regulatory module. A statistical analysis was applied to the pivot, and it was identified as the core pivot.

## Results

### The DEGs in AMI

4551 DEGs (Additional file 1: Table S1) was screened from DEGs analysis. It was observed that the expression characteristics of this group of DEGs. It should be noted that the change in SCD is higher, which may be related to AMI directly or indirectly. Moreover, it may have essential regulatory functions in the development of the disease. While, the analysis of DEGs expression in AMI still needs further study.

### Co-expression behavior of genes associated with AMI

**Fig. 1:**
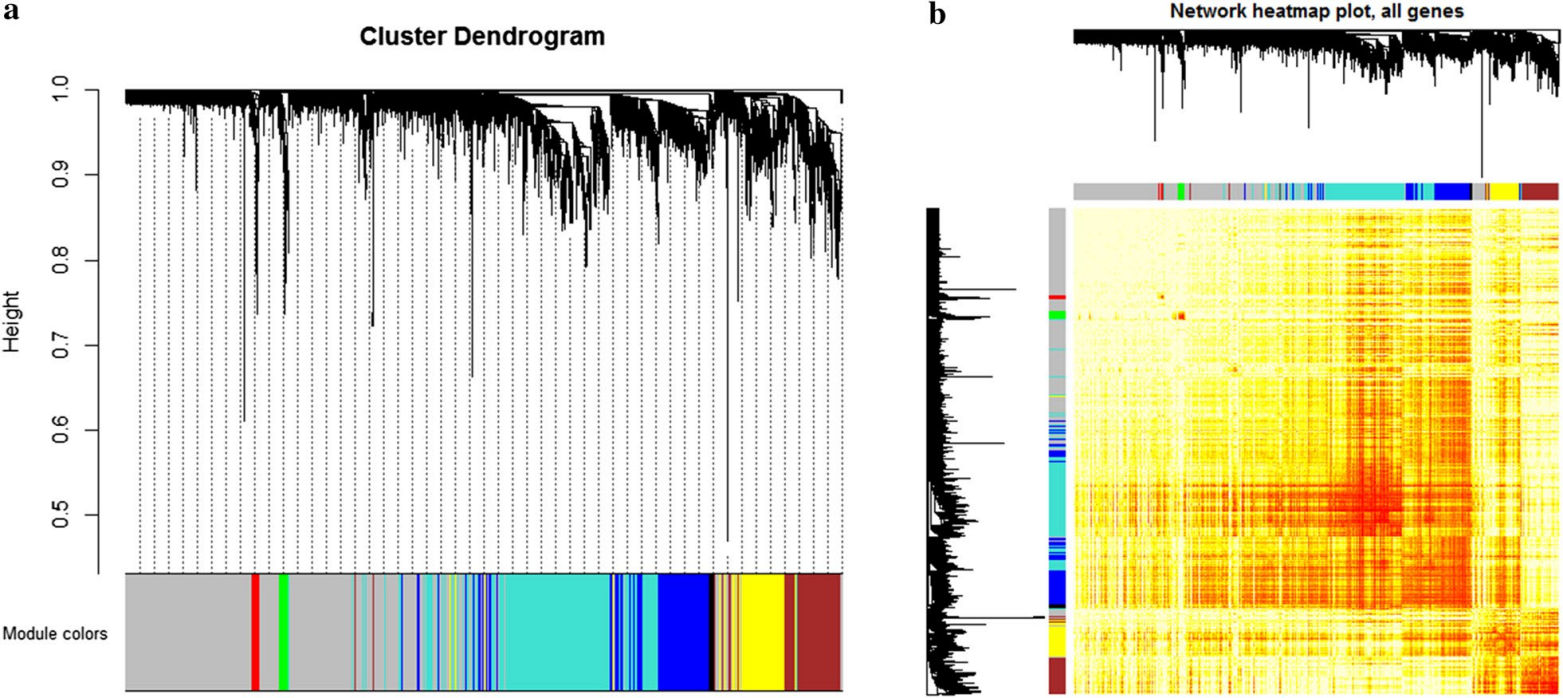
Synergistic expression relationship clustering module for acute myocardial infarction related genes. **a** Clustering into seven modules according to the differential gene cooperative expression relationship, one color indicates a module. **b** Heat map of the expression of the module gene in the sample. A phenomenon of group expression is visually presented in a disease sample of acute myocardial infarction

To study the mechanism of action of related genes in disease samples of patients with AMI systematically, extensive analysis was conducted. Expression profiles of 4551 DEGs in AMI patient samples were constructed. Based on the co-expression network analysis with the gray module removed, it was obtained that the expression of 7 groups of AMI-related disorders. These 7 sets of imbalance modules were identified as dysfunction modules, the DEGs contained within these modules all had a cooperative expression behavior (Fig. 1). These dysfunction modules might be involved in different functions and pathways, indicating different regulatory mechanisms that may mediate the development of AMI.

### Dysfunction modules characterize the pathogenesis of AMI

**Fig. 2:**
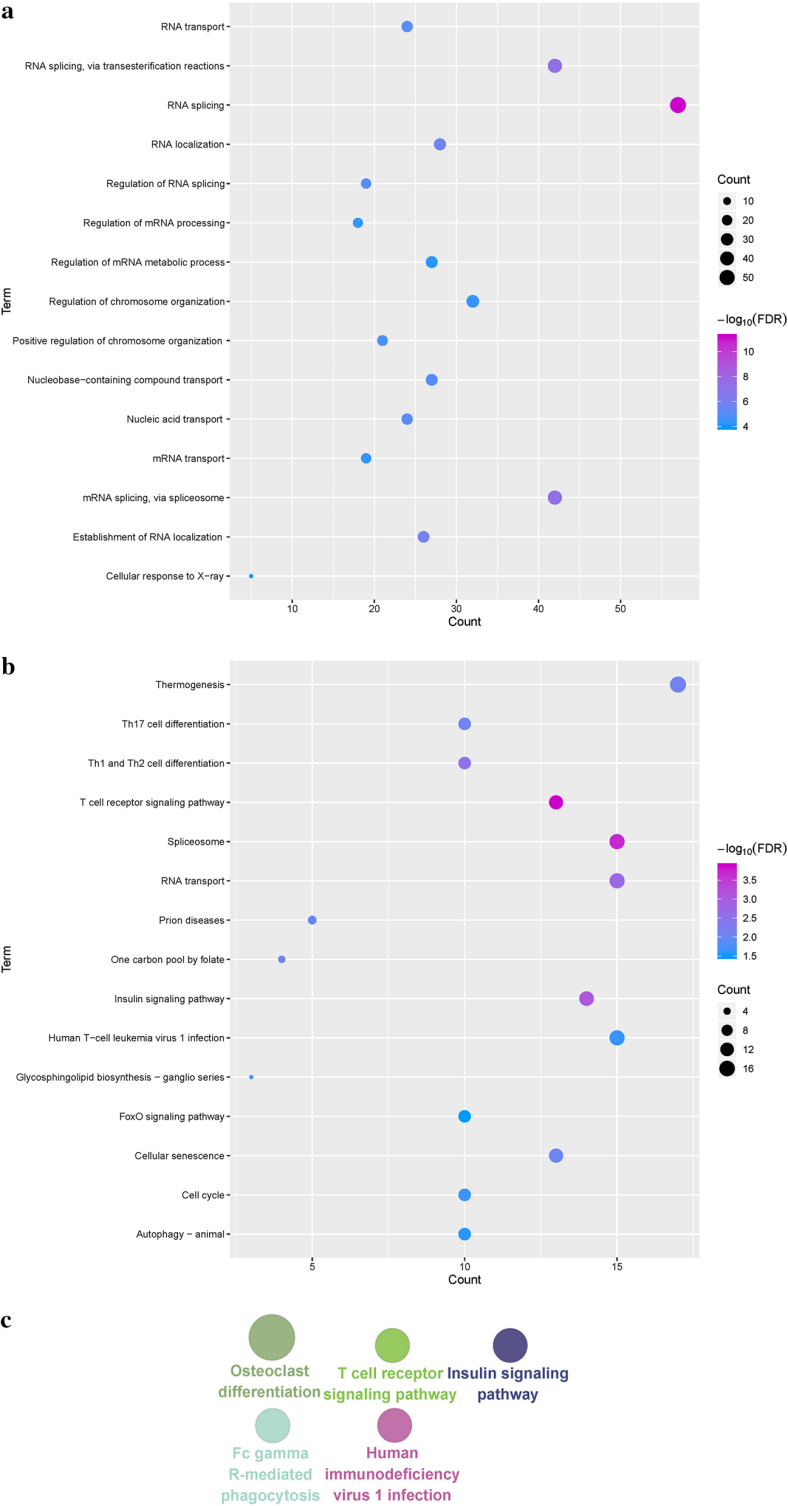
Functional and pathways involved in modular gene identification of dysfunction modules for acute myocardial infarction. **a** Module gene GO function enrichment analysis excerpt. The deeper the color, the stronger the enrichment. The larger the circle, the more critical the proportion of the gene in the module that accounts for the GO function. **b** Module gene KEGG pathway enrichment analysis excerpt. The deeper the color, the stronger the enrichment. The larger the circle, the more critical the proportion of the gene in the KEGG pathway entry. **c** Corresponding functional and access networks

Exploring the functions and pathways involved in DEGs is an essential mean for identifying their pathogenesis. To study the possible dysfunction caused by modular gene imbalance, GO functions and KEGG pathways enrichment analysis on 7 modules were performed. A wealth of GO terms, including 2677 cell composition (CC) entries, 4274 molecular functional (MF) terms, and 22975 biological processes (BP) was collected (Fig. 2a, Additional file 2: Table S2). Based on functional enrichment analysis, it was observed that relevant functional modules tend to involve in multiple disease-related functions, such as prostaglandin, transport of isoprenoid, metabolic process, regulation of DNA recombination, and nucleobase-containing compound transport. On the other hand, 1148 KEGG pathway enrichment results (Fig. 2b) reflect that the functional module genes are mainly involved in the AMP-activated protein kinase (AMPK) signal transduction, phosphatidylinositol 3 kinase**-**serine threonine kinase(PI3K-Akt) signal transduction, and T cell receptor signal transduction. Since the functional and pathway results of modular gene enrichment were strictly related to AMI, the 7 modules were identified as dysfunction modules. From the above data, it can be found that the AMPK signal transduction may be closely related to the induction of AMI. Modular genes can regulate a range of functions and pathways, and module dysregulation is likely to be an essential cause of morbidity. Based on the relationships between the modules, the corresponding function and access networks were built, and identified the proportion of modules involved in the corresponding functions and pathways (Fig. 2c). This might be the dysfunctional global mechanism of AMI. The dysregulation of genes of the module can trigger dysfunction of the module, which in turn affected the functions and pathways involved, and it guided to the occurrence and progression of the disease.

### The ncRNA and TFs driving acute myocardial infarction

**Fig. 3:**
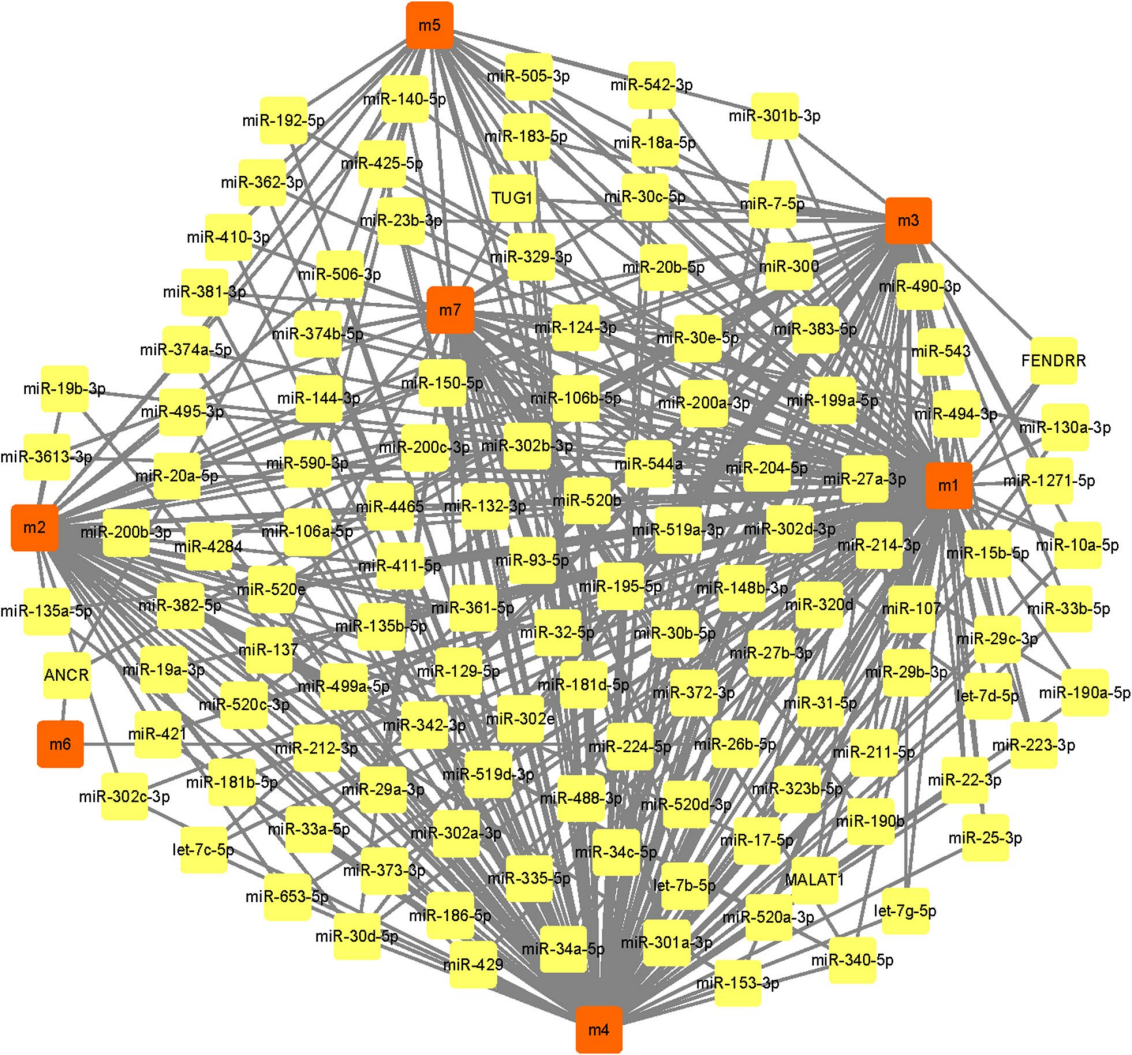
ncRNA regulatory network map of acute myocardial infarction. The orange square indicates the module, and the yellow square indicates the ncRNA corresponding to the module

**Fig. 4:**
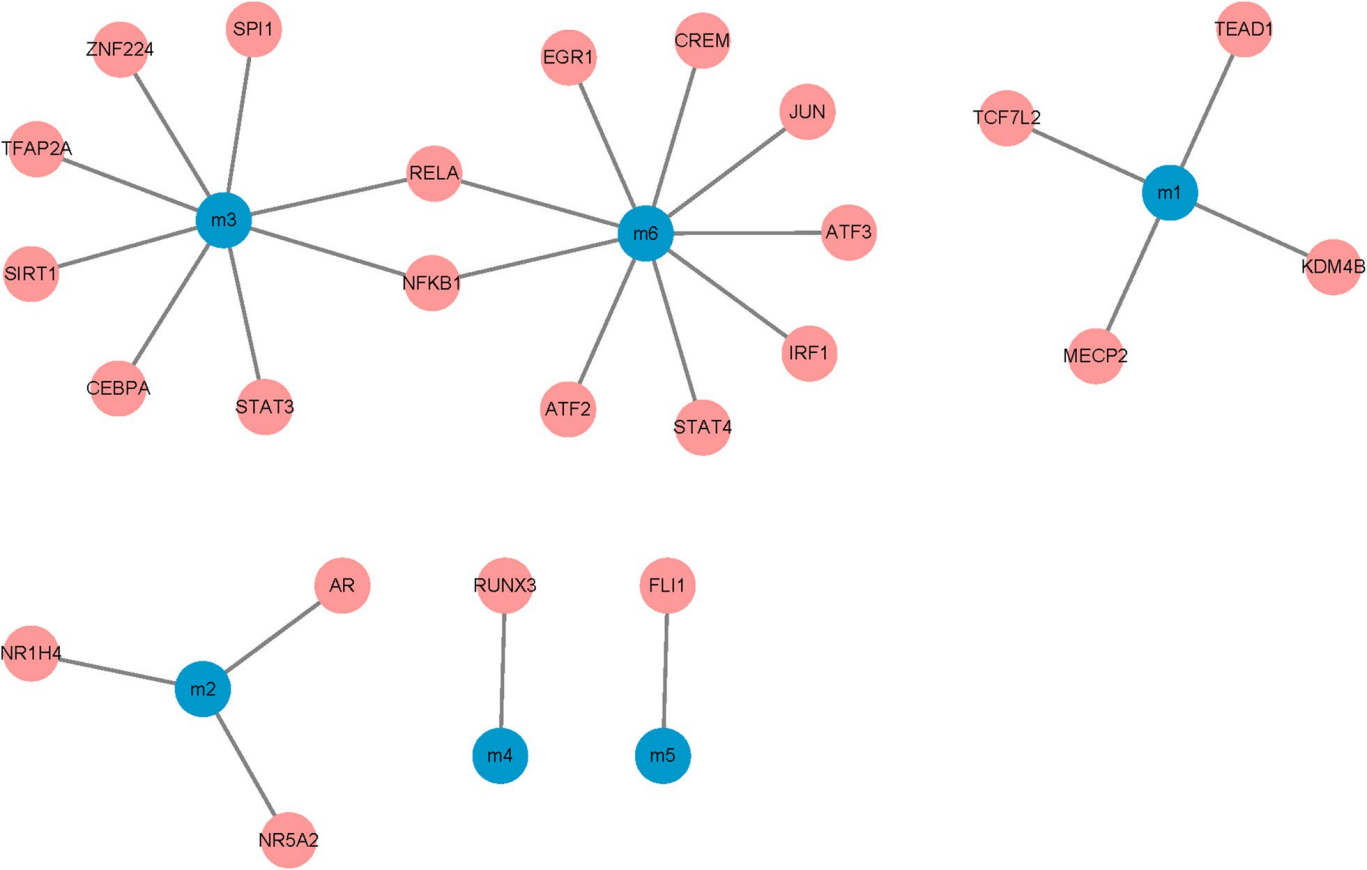
Regulatory network map of transcription factors for acute myocardial infarction. The blue circle indicates the module, and the pink circle indicates the transcription factor corresponding to the module

The transcription and post-transcriptional regulation of genes had been recognized as the critical factors, which regulated the development of diseases, and ncRNA was considered to be an essential regulator. Accurate prediction of ncRNAs that can regulate dysfunction module genes can facilitate the in-depth exploration of the transcriptional regulatory mechanisms of AMI. The ncRNA regulators were explored according to cpRNA-based pivot analysis, which can cause dysfunction of the module. The predicted conclusion (Fig. 3, Additional file 3: Table S3) showed that 782 ncRNA had significant regulatory effects on the module, involving 1239 ncRNA-module interaction pairs. These ncRNAs affected the development of AMI in varying degrees. The analysis found that miR-519d-3p had crucial regulatory functions in 6 dysfunction modules, which affected the progression of AMI. Both TUG1 and miR-93-5p had meaningful regulatory relationships with 4 dysfunction modules and played an important role in module dysfunction. Other ncRNAs exhibited significant modulation of dysfunction modules, and had essential functions in the regulation of acute myocardial infarction. The occurrence and development of diseases were inextricably linked to the imbalance of TFs, which was reflected in the regulation of TFs to dysfunction modules. The pivot analysis was performed to the module based on the regulatory relationship of the TFs and genes. The conclusion showed that (Fig. 4, Additional file 4: Table S4), a total of 24 TFs had crucial transcriptional regulation of the dysfunction module of AMI, which involved 26 TF-Module interaction pairs. Statistical analysis on the regulation of these TFs was performed and it was found that nuclear factor kappa B (NFκB)1 importantly regulated 2 dysfunction modules, thereby promoting the occurrence and development of AMI. Both Methyl-CpG binding protein 2 (MECP2) and silent information regulator 2 homolog 1(SIRT1) had regulatory functions in 1 functional module and occupied an indispensable position in the potential pathogenesis of AMI.

## Discussion

Myocardial infarction is the guiding cause of high mortality in all cardiovascular diseases [32]. Mortality after myocardial infarction has decreased essentially over the past few decades, while there is still important in-hospital mortality [33]. Therefore, research on the pathogenesis and treatment mechanism of AMI has become a top priority. Many biologists and medical researchers have invested in the pathogenesis of AMI. They mainly focused on some certain genes, and some researches had achieved results in proteins and related signal transductions. A series of analytical methods was combined to explore the pathogenesis of AMI. The complete expression profile of AMI disease samples was constructed for DEGs analysis, and 4551 potential pathogenic genes were screened. The increased SCD content indicated that SCD may be the key gene for AMI. The results of the study suggested that the ischemic time from the onset of symptoms to coronary reperfusion appears to be the strongest factor affecting thrombus in myocardial infarction [34]. Co-expression analysis of the DEGs revealed that we obtained 7 co-expression modules, and the genes contained in the modules were considered to have synergistic expression. Based on the results of the functional enrichment analysis, it was found that 7 modules were mainly involved in response to reactive oxygen species. According to studies by many scholars, N-acetylcysteine (NAC) is an antioxidant with active oxygen scavenging properties, which has the effect of enhancing nitroglycerin [35]. The enrichment of pathways revealed that functional block genes were primarily involved in the AMPK signal transduction, which might trigger AMI. This suggested that the AMPK signaling cascade was thought to be the core signal transduction that triggered AMI. Yang et al. [36] accentuated the AMPK signal transduction has key functions in intracellular adaptation to energy stress during myocardial ischemia. Inhibition of AMPK signaling by Notch1 enhances cardiac dysfunction caused by myocardial infarction [36]. The TFs which were involved in these 7 dysfunction modules, were obtained, and there were 26 Pivot-Module interaction pairs. NFκB1 regulated 2 dysfunction modules, thereby promoting the occurrence and development of AMI. According to Boccardi et al. [37], it was found that NFκB was involved in various human diseases, including atherosclerosis and myocardial infarction. Studies had shown that the -94 ins/del ATTG NFκB1 gene variant might lead to a decrease in myocardial infarction sensitivity by a potential reduction in activation of NFκB, which in turn was associated with a decrease in plasma inflammatory markers [37]. Both MECP2 and SIRT1 had regulatory functions in 1 functional module and occupied an indispensable position in the potential pathogenesis of AMI. On the one hand, the anti-apoptotic effect of miR-22 was to protect myocardial infarction by targeting MECP2 [38] directly. On the other hand, SIRT1 was known to be a nicotinamide adenine dinucleotide-dependent histone deacetylase, which makes the heart more resistant to ischemic injury. SIRT1 may be a new promising therapeutic target for myocardial infarction [39]. The expression of SIRT1 was down-regulated by many stress stimuli in the heart. These stimuli might jointly drive the pathogenesis of AMI [40]. Moreover, ncRNA had been recognized as an important regulator in the development and progression of the disease. In this regard, a pivot analysis was performed, based on the targeting relationship between ncRNA and genes. The predicted results showed that 782 ncRNA had important regulatory effects on the modules, involving 1239 ncRNA-Module interaction pairs. These ncRNAs affected the development of AMI in varying degrees. The results of statistical analysis revealed that miR-519d-3p had important regulatory functions in 6 dysfunction modules, which might affect the progression of AMI. Down-regulation of miR-519d-3p and over expression of HOX transcript antisense RNA(HOTAIR) had been reported reducing myocardial apoptosis induced by myocardial infarction or hypoxia. It can provide a potential therapeutic target for myocardial infarction [41]. Both TUG1 and miR-93-5p had essential regulatory relationships with the 4 dysfunction modules, and had important functions in module dysfunction. On the one hand, TUG1 can inhibit apoptosis in hypoxia-induced injury of H9c2 cell, thereby reducing hypoxia-induced cell damage and inhibiting myocardial infarction [42]. On the other hand, the data showed that the expression of miR-93-5p had a cardioprotective effect in AMI. The Adipose-derived stromal cells (ADSCs)-derived exosomes enhanced by miR-93-5p could prevent cardiac damage by inhibiting autophagy and inflammatory responses [43]. The series of regulatory factors predicted by this study had a certain degree of regulation on the pathogenesis of AMI.

Actually, there are many other mechanisms underlying AMI. Adiponectin and insulin resistance play a critical role in progression of any stage of ischemic heart disease [44]. Tight glycemic control may improve outcome of ST-segment elevation myocardial infarction and increase regenerative potential of the ischemic myocardium after acute myocardial infarction [45, 46]. The potential interplay between subclinical hypothyroidism and inflammatory activity makes atherosclerotic plaque progression toward instability, resulting in the innate immunity-dependent plaque rupture [47]. Ubiquitin–proteasome deregulation is proposed as the pathogenic factor mediating the progression of the plaque [48] and Myocardial carbonic anhydrases activation is associated with ischemic cardiomyopathy in diabetic patients [49]. However, except for the key factors which are above-mentioned, other unmentioned factors might have functions in the mechanism of dysregulation of AMI, which need to be further explored.

### Limitations

The results of this study are based solely on bioinformatics models. No further in-depth studies were conducted to verify the results of this study, such as cell experiments, animal experiments, human specimen experiments and so on.

## Conclusions

Overall, it was indicated that the core key gene, SCD, was the responsible for the development of AMI. It not only provided a new way for biologists and pharmacologists to study the effects of AMPK signaling on myocardial infarction, but also provided a valuable reference for their subsequent treatment options.

## Supplementary Information

**Additional file 1: Table S1.** Differentially expressed genes.

**Additional file 2: Table S2. **Functions and signaling pathways of modular gene involvement.

**Additional file 3: Table S3. **NcRNA pivot that regulates module genes.

**Additional file 4: Table S4.** TF pivot that regulates module genes.

## Data Availability

PRJNA208840.

## Abbreviations

AMI: Acute myocardial infarction
SCD: Stearoyl-CoA desaturase
DEGs: Differentially expressed genes
NFκB: Nuclear factor kappa B
AMPK: AMP-activated protein kinase
